# RiMS-FiLM: Non-Destructive Multimodal Screening for Fixed-Budget Re-Inspection Prioritization of Maize Kernels

**DOI:** 10.3390/foods15193400

**Published:** 2026-09-24

**Authors:** Mingwen Bi, Ziqian Zhang, Xiaobo Yang, Feng Li, Ge Gao, Jianlei Kong, Min Zuo, Qingchuan Zhang

**Affiliations:** 1 Business School, Beijing Wuzi University, Beijing 101149, China; bimingwen@bwu.edu.cn (M.B.);; 2 Beijing Key Laboratory of Smart Supply Chain Application and Risk Management, Beijing Wuzi University, Beijing 101149, China; 3 National Engineering Research Center for Agri-Product Quality Traceability, Beijing Technology and Business University, Beijing 100048, China

**Keywords:** grain quality assessment, machine vision, physical attributes, selective screening, prototype learning, multimodal representation

## Abstract

Rapid, non-destructive prioritization of maize kernels can help allocate limited re-inspection capacity in post-harvest quality control. Existing deep learning approaches mainly optimize overall classification accuracy and do not explicitly address which samples should be inspected first when verification resources are constrained. This study proposes RiMS-FiLM, an end-to-end multimodal screening framework that integrates kernel RGB appearance with lightweight physical attributes (weight and size) to generate re-inspection priority scores. Feature-wise Linear Modulation adaptively conditions visual representations on physical measurements, while a prototype-guided module organizes fused embeddings into class-structured operational quality-priority regions. The risk tiers are derived from the original GrainSet-Maize defect categories and are used as screening-priority labels rather than laboratory-validated toxicological endpoints. Internal evaluation on the stratified split of the single public GrainSet-Maize dataset over five random seeds yielded a Macro-F1 of 0.9917 ± 0.0027 and captured 94.65 ± 0.21% of high-priority samples within a 20% re-inspection budget. It also reduced severe high-to-low priority errors compared with representative fusion and selective-screening baselines. These results demonstrate the potential of low-cost multimodal sensing for resource-constrained, non-destructive grain quality screening and re-inspection planning.

## 1. Introduction

In large-scale grain storage and purchasing, throughput, inspection costs, and labor availability constrain the number of kernels that can be inspected in detail. Low-cost, non-destructive screening based on machine vision and basic physical measurements can process large numbers of samples rapidly, whereas more intensive re-inspection is limited by the associated time, cost, and labor requirements [1,2,3,4]. Therefore, a practical inspection system should not only predict quality labels, but also determine which kernels should be inspected first. Under a fixed re-inspection budget, the operational objective is to rank abnormal or high-priority kernels near the top of the screening list so that limited verification capacity is allocated efficiently.

Recent advances in machine vision, deep learning, and non-destructive sensing have substantially improved automated cereal-quality assessment. Convolutional neural networks [5], visual Transformers [6], and non-destructive imaging methods [7,8] have shown strong performance in detecting kernel damage, discoloration, pest attack, and other visible defects. Beyond RGB appearance, complementary sensing channels such as hyperspectral or near-infrared measurements and lightweight physical attributes can provide additional information on composition and structural condition, supporting more robust quality assessment across heterogeneous samples [9,10].

Most existing approaches still optimize classification accuracy or defect-identification performance and implicitly treat samples as equally important, without considering the practical constraint of limited re-inspection resources [11,12]. Under a fixed budget, however, screening utility depends on whether high-priority kernels are ranked early enough to enter the restricted re-inspection subset [13,14].

Also, the complexity of risk priority judgment is increased by the multimodal nature of low-cost screening cues. The visual appearance principally indicates phenomena like mold growth, surface discoloration, and pest activity, whereas lightweight physical characteristics might reveal the loss of integrity, degrees of deformation, and associations with impurities which cannot be readily observed with the naked eye. Despite the popularity of multimodal modeling in the field of agricultural perception, the current literature mostly assumes that all modalities are equally reliable and tries to diminish the differences between modalities during the fusion process [15,16]. However, in practice, intermodal consistency highly depends on specific sample conditions. An example is that the appearance of some grains with normal appearance may show abnormal weight or size variations simultaneously because of shooting angles or lighting. These are not data collection noise but can often correspond to potential quality risks [17]. When the sample-level modality reliability is not constant and cross-modal conflict structures cannot be explicitly modeled, the ability of the model to remain stable and to discriminate will be significantly affected in the risk ranking task.

Alongside this, the two-stage pipeline modeling paradigm is also widely used in current research on quality management of cereals such as risk stratification: firstly, quality groups or pseudo-labels are constructed using unsupervised clustering or heuristic rules, and then independent prediction models are fitted to the grouping outcomes [18,19,20]. Even though this strategy is convenient for establishing hierarchical division, its operation is extremely reliant on the stability of the initial grouping, and the representation learning procedure is separated from the downstream screening decision, readily introducing the issue of error accumulation and structural mismatch [21,22]; thus, it is not simple to establish a stable and budget-aware multi-level risk ranking framework.

To respond to these difficulties, this paper introduces a multimodal end-to-end risk screening framework, RiMS-FiLM, that aims to prioritize the discovery of high-risk samples under a fixed re-inspection budget instead of merely pursuing traditional classification accuracy. This framework forms the multimodal fusion process upon the Feature-wise Linear Modulation (FiLM) mechanism that adaptively modulates the visual features using the physical properties at the sample level and integrates conflict-aware modeling strategies to explicitly depict cross-modal inconsistencies, and creates a representation space that is more suitable for the needs of risk screening tasks. It is based on this that a prototype-guided risk structure learning mechanism is presented to arrange multimodal embeddings into relatively compact and separable low-, medium-, and high-priority regions, to allocate multimodal embeddings by stable priority ranking without post hoc clustering. The model directly outputs continuous risk scores to serve real-world screening decisions through a unified end-to-end optimization framework. Extensive experimental results using the GrainSet-Maize multimodal maize kernel dataset reveal that RiMS-FiLM improves the high-risk discovery rate under limited re-inspection capacity constraints, outperforming traditional two-stage hierarchical methods and representative selective screening paradigms. Accordingly, the methodological contribution of RiMS-FiLM lies in adapting and integrating these established components into an end-to-end fixed-budget re-inspection prioritization framework for maize kernels.

The primary contributions of this work can be outlined as follows:

(1) We define the quality inspection of maize kernels as a risk screening problem under a fixed re-inspection budget constraint, shifting the evaluation focus from global classification accuracy to decision-oriented screening effectiveness under real re-inspection capacity constraints, making the model optimization objective consistent with the real quality management needs.

(2) We present a FiLM-based multimodal fusion mechanism and introduce a conflict-aware modulation strategy to capture the sample-dependent modality reliability variations and also the cross-modal discrepancies between visual features and physical attributes, hence learning risk-aware representations which are more suitable for the risk priority ranking task.

(3) We design an end-to-end joint optimization framework that jointly learns multimodal embed-ding representations, prototype-guided structured risk representations, and continuous risk scores to make screening decisions, achieving stable and interpretable priority ranking without staged clustering.

## 2. Materials and Methods

### 2.1. Materials: Dataset and Risk Annotation

#### 2.1.1. Grainset Maize Subset and Multimodal Fields

The GrainSet-Maize dataset is a publicly available benchmark comprising approximately 19,000 maize kernel samples with high-resolution RGB images and associated metadata [23]. The metadata include defect labels, per-kernel size, batch-associated physical weight, collection time, and coarse location information. In this study, only RGB images, weight, and size were used as model inputs; time and location were retained solely for split-distribution diagnostics (Table 1). These measurements provide low-cost, non-destructive cues for operational grain-quality screening. All RGB images and metadata used here were taken directly from GrainSet-Maize; no kernels were re-imaged or physically re-measured by the authors. In the released annotations, size is a per-kernel area measurement (mm^2^), whereas weight is a batch-associated physical-weight field (g); GrainSet forms batches within DU categories using 1 mg weight intervals. In the maize subset, 484 distinct weight values occur, with each value shared by 1–257 kernels (median 20.5; mean 39.3). RGB appearance captures visible surface defects, while size and weight provide complementary low-cost physical cues.

#### 2.1.2. Defect Categories and Operational Risk Stratification

The GrainSet-Maize subset contains eight quality categories: Normal (NOR), Fusarium & Shriveled (F&S), Sprouted (SD), Moldy (MY), Attacked by Pests (AP), Broken (BN), Heat Damaged (HD), and Impurities (IM) [23]. Representative examples are shown in Figure 1.

Table 2 summarizes the dataset distribution as defined by the original dataset creators. The original dataset has predefined training datasets and this table provides the number of images for each of the eight categories of defects for both of the dataset splits. This table serves as an overview of the dataset structure under its original definition and does not reflect the risk stratification scheme introduced in Section 2.1.3.

Different defect categories can imply different degrees of storage-quality concern and therefore different priorities for re-inspection. In practical maize storage management, kernels exhibiting mold-related symptoms, fungal infection, sprouting, pest damage, or other visible abnormalities may warrant earlier verification because these conditions are more closely associated with storage-quality deterioration or processing-related defects than visually normal kernels. Accordingly, the risk tiers used in this study represent operational screening priorities for storage-quality management rather than laboratory-validated toxicological or food-safety risk levels. The mapping used here is therefore an operational re-inspection rule, guided by the defect definitions in GrainSet and storage-quality considerations reported in related grain-grading studies [18,19,20]: F&S, MY, SD, and AP are assigned earlier re-inspection priority because they indicate biological deterioration or active damage; BN, HD, and IM are assigned medium priority as integrity/processing-related defects; NOR is assigned low priority. These tiers do not imply equivalent toxicological severity within a tier.

Following this operational rationale, the study re-categorizes the original eight defect classes into three operational quality-risk tiers used as re-inspection-priority screening labels: NOR is low risk; IM, BN, and HD are medium risk; and F&S, SD, MY, and AP are high risk (Table 3).

#### 2.1.3. Risk-Stratified Data Split Protocol

To ensure a reliable evaluation of the algorithm’s screening capability across risk levels, a risk-stratified sampling strategy is employed, distributing samples into training, validation, and test sets at a ratio of 7:1:2. This approach ensures adequate representation of each risk level during training, thereby avoiding performance assessment bias arising from imbalanced sample sizes across risk tiers. The 70% training subset is used for parameter estimation, the 10% validation subset for model selection and early stopping, and the remaining 20% is kept as an internal test set.

In order to illustrate the statistical consistency of the risk-stratified split strategy, the training, validation, and testing sample distributions were compared for defect category, acquisition time, and two physical attributes. As shown in Figure 2, the three splits all exhibited highly similar label proportions, temporal coverage, and kernel weight and size distributions; these comparisons describe marginal distribution consistency but do not by themselves establish sample independence.

Because the released per-kernel XML does not contain an explicit physical-batch identifier, exact batch membership cannot be reconstructed. As a conservative robustness check, we therefore constructed a grouped split using (DU_grain, weight) as the grouping key, because kernels from the same GrainSet batch share the same DU category and weight annotation. All 1920 such groups were assigned exclusively to one of the training, validation, or test partitions (13,301/1900/3799 samples), with zero group overlap across partitions. This grouped protocol is used only as an additional robustness evaluation; the stratified 7:1:2 split remains the primary evaluation protocol.

#### 2.1.4. Data Preprocessing and Normalization

RGB images were resized to 224 × 224 pixels using bilinear interpolation. During training, random horizontal flipping was applied with probability 0.5, and ColorJitter (brightness = 0.2, contrast = 0.2, saturation = 0.2, hue = 0.1) was applied with probability 0.3. Images were then normalized using the ImageNet channel mean (0.485, 0.456, 0.406) and standard deviation (0.229, 0.224, 0.225).

To address scale differences across modalities and prevent data leakage, physical features are normalized using z-score statistics computed solely on the training set and then fed into the physical feature encoder described in Section 2.4.2.

These normalization steps reduce scale differences across modalities and improve numerical stability during end-to-end optimization; they do not guarantee invariant results.

### 2.2. Problem Formulation: Fixed-Budget Multimodal Risk Screening

#### 2.2.1. Screening-to-Verification Setting and Notation

Let $D={\left\{(x_{i},p_{i},y_{i})\right\}}_{i=1}^{N}$ denote a maize kernel dataset with multimodal features, where $x_{i}\in R^{H\times W\times 3}$ denotes the RGB image of the *i*-th kernel, $p_{i}\in R^{d_{p}}$ denotes the physical features consisting of kernel weight and size, and $y_{i}$ denotes the risk label—low risk, medium risk, or high risk—corresponding to the three risk tiers mapped from the eight defect categories. In this study, “risk” denotes operational re-inspection priority derived from the defect categories rather than a laboratory-validated toxicological safety endpoint.

In real grain storage quality inspection, due to limited inspection resources, only a fraction of samples can enter the re-inspection stage. Therefore, we should first use multimodal features to perform low-cost screening of maize kernels. Formally, let B∈(0,1] denote the re-inspection budget ratio, representing the maximum proportion of kernels that can enter the re-inspection stage. The corresponding number of kernels selected for re-inspection can be expressed as $K=\left\lceil B\cdot N\right\rceil$.

The screening model f(⋅) maps each kernel’s multimodal input $\left(x_{i},p_{i}\right)$ to a continuous risk score $s_{i}=f(x_{i},p_{i})\in R$, where a higher value indicates a higher priority for re-inspection. The screening decision selects the top−K kernels based on the ranked predicted risk scores:

(1) $$\pi_{B}=TopK({\left\{s_{i}\right\}}_{i=1}^{N},K)$$

Therefore, the objective of the fixed-budget multimodal risk screening proposed in this paper is, under limited re-inspection conditions, to design a f(⋅) such that the selected $\pi_{B}$ can cover as many high-priority maize kernels as possible.

#### 2.2.2. High-Risk Prioritization Objective

Traditional grain classification algorithms are mostly modeled with the objective of minimizing the overall prediction error across all samples. In operational maize storage screening, however, the focus is on selecting as many high-priority kernels as possible into the re-inspection subset. Therefore, we define $y_{i}^{H}\in \{0,1\}$ to indicate whether the *i*-th kernel belongs to the high-risk level.

The screening objective is to maximize the number of high-risk kernels retrieved in $\pi_{B}$, which can be formally expressed as $\max_{f}\sum_{i\in \pi_{B}}y_{i}^{H}$. While utilizing a fixed budget constraint, optimizing for rank quality as opposed to classifying correctly gives the model incentive to have kernels assigned higher re-inspection priority to be ranked earlier on the screening list, which results in effective resource use in real-world physical storage situations.

#### 2.2.3. Budget-Aware Evaluation Metrics

We adopt several popular budget-aware metrics to quantitatively evaluate screening effectiveness.

The high-risk recall at budget B is defined as

(2) $${Recall}_{H}@B=\frac{\sum_{i\in \pi_{B}}y_{i}^{H}}{\sum_{i=1}^{N}y_{i}^{H}}$$

The metric is the proportion of the high-risk kernels that were captured in a selected re-inspection subset. When B=1, all samples are selected for re-inspection and the ranking component becomes operationally irrelevant; this limiting case does not make the ranking objective equivalent to multiclass classification. In practice, B≪1, and screening effectiveness thus depends primarily on the early retrieval of high-risk samples.

Correspondingly, the proportion of missed high-risk samples is defined as

(3) $${Missed}_{H}@B=1-{Recall}_{H}@B$$

High-risk kernels that are undetected under a specific budget are measured by this metric.

Because the reported operational budget range is limited to 0–30%, we use the partial area under the missed-high risk–coverage curve ($p{AURC}_{0.30}$) as the primary integrated screening metric:

(4) $$p{AURC}_{0.30}=\int_{0}^{0.30}{Missed}_{H}(B)dB$$

A lower $p{AURC}_{0.30}$ indicates faster discovery of high-priority kernels within the operational range. It is computed from all attainable sample-level budget points over B∈[0, 0.30].

### 2.3. RiMS-FiLM: End-to-End Multimodal Risk Screening Framework

The RiMS-FiLM framework proposed in this paper is an end-to-end multimodal risk screening system that directly maps low-cost kernel observations to screening-oriented risk scores under a fixed re-inspection budget. As shown in Figure 3, the multi-task learning-based RiMS-FiLM framework jointly optimizes multimodal representation learning, conflict-aware fusion, structured risk space modeling, and screening-oriented risk prediction during training.

Given a maize kernel sample $\left(x_{i},p_{i}\right)$, where $x_{i}$ denotes the RGB image features and $p_{i}$ denotes the physical features, RiMS-FiLM first extracts a representation from each modality using dedicated encoders. These representations are then adaptively fused through the FiLM-based conflict-aware modulation mechanism to generate the joint multimodal embedding $z_{i}$. A prototype-guided regularizer subsequently imposes class-structured constraints on $z_{i}$. Finally, the prediction head outputs class probabilities from which the screening score $s_{i}$ is obtained for fixed-budget prioritization as defined in Section 2.2.

#### 2.3.1. End-to-End Multimodal Risk Learning

Traditional staged hierarchical methods treat representation learning and downstream screening decisions as independent subtasks completed separately. This leads to a lack of consistency constraints across subtasks during training, and the performance of such pipeline paradigms is entirely dependent on static intermediate results, making them highly susceptible to error accumulation. RiMS-FiLM, through end-to-end multi-task joint training, achieves collaborative optimization of multimodal representation, conflict modeling, and risk ranking, enabling the model to learn more discriminative risk representations under unified objective constraints.

Formally, the multimodal encoders first map image features $x_{i}$ and physical features $p_{i}$ into task-specific vector spaces: $v_{i}=g_{v}\left(x_{i}\right),\;u_{i}=g_{p}\left(p_{i}\right)$, where $g_{v}\left(\cdot \right)$ and $g_{p}\left(\cdot \right)$ denote the visual and physical encoders, respectively. The multimodal representation $z_{i}$ is obtained through the proposed fusion module: $z_{i}=\Phi (v_{i},u_{i})$ (see Section 2.5). The prototype-guided objective then regularizes $z_{i}$ relative to learnable class prototypes $\mu_{k}$, while the prediction head converts $z_{i}$ into class probabilities and the screening score $s_{i}$ used for fixed-budget prioritization.

#### 2.3.2. Structured Risk Representation and Decision Alignment

A key design principle of RiMS-FiLM is to align representation learning with screening decision objectives through structural constraints on risk levels. Unlike traditional methods that rely on implicitly learned classification boundaries, this framework uses a prototype-guided mechanism to form structure distributions corresponding to risk levels in the embedding space, enabling samples with similar risk levels to cluster around their corresponding prototypes, thereby improving the effectiveness and interpretability of screening decisions.

Let $\mu_{L},\mu_{M},\mu_{H}$ denote the learnable prototypes corresponding to the low-, medium-, and high-priority classes. For a sample representation $z_{i}$, the prototype objective reduces the distance to the target prototype while enforcing a margin from the nearest non-target prototype. This encourages intra-class compactness together with explicit separation from competing class prototypes, providing a class-structured representation for downstream screening.

### 2.4. Multimodal Encoders

#### 2.4.1. Visual Encoder for Maize Kernel Appearance

Kernel images are processed using a dedicated visual encoder $g_{v}(\cdot )$ to obtain appearance-based feature representations. For each input image $x_{i}$, the encoder outputs a visual embedding:

(5) $$v_{i}=g_{v}\left(x_{i}\right),\;v_{i}\in R^{d}$$

In this work, the Swin Transformer (Swin-T) architecture pretrained on ImageNet is adopted as the visual backbone [24]. The hierarchical transformer structure provides multi-scale feature representations suitable for capturing surface-level variations in kernel appearance.

#### 2.4.2. Physical Encoder for Lightweight Kernel Attributes

The two physical metadata fields used by the model—normalized batch-associated weight and per-kernel size—are processed through a separate encoder. The physical encoder maps the low-dimensional attribute vector into the same latent feature space as the visual embedding:

(6) $$u_{i}=g_{p}\left(p_{i}\right),\;u_{i}\in R^{d}$$

The encoder is implemented as a multilayer perceptron composed of fully connected layers with GELU nonlinear activations and dropout. This design projects physical attributes into aligned feature representations that can be directly combined with visual embeddings in subsequent fusion modules.

### 2.5. Conflict-Aware FiLM Fusion

Multimodal fusion strategy plays a crucial role in risk-aware representation learning. Traditional multimodal fusion strategies mostly follow the implicit assumption that all modalities are consistently reliable, and directly employ concatenation or simple weighted averaging to exploit the complementary advantages of multimodal features. However, in the maize kernel quality inspection task, the visual and physical modalities often provide inconsistent cues. For example, a kernel may appear visually intact but exhibit abnormal weight or size due to internal deterioration. To address this, this paper proposes a FiLM-based conflict-aware fusion mechanism to adaptively regulate cross-modal interactions at the feature level.

#### 2.5.1. Film-Based Cross-Modal Modulation Formulation

Given the visual embedding $v_{i}\in R^{d}$ and the physical embedding $u_{i}\in R^{d}$ obtained from Section 2.4, the proposed fusion mechanism modulates the visual representation conditioned on the physical features. To keep the forward path consistent with the implemented model, the FiLM operation and the subsequent layer normalization are expressed jointly as

(7) $$z_{i}=LN(\gamma \left(u_{i}\right)⨀v_{i}+\beta (u_{i}))$$

where $\gamma \left(\cdot \right)$ and β(⋅) are learnable transformation functions implemented as lightweight fully connected layers, ⨀ denotes element-wise multiplication, and LN denotes layer normalization. No additional post-FiLM concatenation is used in the reported implementation.

The functions $\gamma \left(u_{i}\right)$ and $\beta (u_{i})$ produce feature-wise scaling and shifting coefficients, enabling adaptive reweighting of visual channels according to physical cues. The layer-normalized representation $z_{i}$ is then passed to the classifier to obtain the base logits $l_{i}=W_{c}z_{i}+b_{c}$.

In parallel, the implementation explicitly quantifies cross-modal discrepancy in the shared embedding space as $c_{i}=1-cos(v_{i},u_{i})$. The scalar discrepancy score $c_{i}$ is passed through a learnable two-layer MLP $h_{c}(\cdot )$. Its output is used to refine the high-priority logit, while the low- and medium-priority logits remain unchanged:

(8) $${\tilde{l}}_{i,H}=l_{i,H}+\alpha h_{c}(c_{i}),{\tilde{l}}_{i,L}=l_{i,L},{\tilde{l}}_{i,M}=l_{i,M}$$

where α=1.0 controls the strength of the conflict-based logit adjustment. This connects the conditional FiLM modulation and the explicit discrepancy estimate within the same forward path: FiLM adapts the visual representation according to the physical cues, whereas the conflict head provides an additional adjustment based on the measured cross-modal discrepancy. No separate conflict-specific auxiliary loss is introduced; the conflict head is optimized end-to-end through the classification objective.

#### 2.5.2. Conflict-Aware Interpretation

The FiLM modulation mechanism described above enables the visual representation to be conditioned on the physical attributes rather than fused by passive concatenation. When the two modalities provide compatible cues, the learned scaling and shifting functions can preserve or emphasize visual channels that are useful for the classification objective. This interpretation reflects the functional role of conditional feature modulation and does not assume that every individual channel has a directly observable semantic meaning.

When the modalities provide discrepant cues, the same conditional modulation pathway allows the visual representation to be adjusted as a function of the physical embedding. The explicit discrepancy branch in Equation (8) additionally modifies the high-priority logit according to the measured cross-modal discrepancy. Together, these two pathways provide a mechanism for sample-dependent multimodal adjustment without requiring a separate conflict-specific loss.

FiLM differs from attention-based fusion in that the physical embedding acts as a conditioning signal that produces feature-wise scale and shift parameters for the visual representation. In RiMS-FiLM, this conditional modulation is complemented by the explicit discrepancy score defined in Section 2.5.1. The intended role is therefore to reduce dependence on a fixed fusion rule when visual and physical cues differ, rather than to treat the physical attributes as a direct measure of visual reliability.

#### 2.5.3. Fusion Variants for Comparative Analysis

To systematically evaluate the contribution of conflict-aware FiLM modulation, several alternative multimodal fusion strategies are implemented as comparative base-lines while keeping the same encoders and training protocol. For these alternatives, $r_{i}$ denotes the variant-specific fusion output; the same final layer normalization is then applied as $z_{i}=LN(r_{i})$ before classification.

(1) Feature concatenation (Concat)

The visual and physical embeddings are directly concatenated and projected into a unified latent space through a multilayer perceptron [25]:

(9) $$r_{i}=\emptyset_{c}(\left[v_{i};u_{i}\right])$$

where [⋅;⋅] denotes vector concatenation and $\emptyset_{c}(\cdot )$ represents an MLP with nonlinear activation and dropout for dimensionality reduction and feature interaction.

(2) Cross-attention fusion

To explicitly model directional modality interaction, visual embeddings serve as query vectors while physical embeddings act as key and value vectors in a multi-head attention mechanism [26]:

$$Q_{i}=v_{i}W_{Q},\;K_{i}=u_{i}W_{K},\;V_{i}=u_{i}W_{V},$$

$$a_{i}=MHA\left(Q_{i},K_{i},V_{i}\right),$$

(10) $${\hat{r}}_{i}=LN\left(v_{i}+a_{i}\right),\;r_{i}={\hat{r}}_{i}+FFN({\hat{r}}_{i})$$

where MHA denotes multi-head attention, LN represents layer normalization, and FFN is a feed-forward network with residual connection.

(3) GatedSum fusion

A complementary gating mechanism is adopted to adaptively balance visual and physical contributions [27]:

$$g_{i}=\sigma (\emptyset_{g}(u_{i})),\;u_{i}^{\prime }=\emptyset_{p}(u_{i})$$

(11) $$r_{i}=g_{i}⨀v_{i}+(1-g_{i})⨀u_{i}^{\prime }$$

where $\emptyset_{g}(\cdot )$ and $\emptyset_{p}(\cdot )$ are MLPs, σ(⋅) denotes the sigmoid function, and ⨀ indicates element-wise multiplication.

These fusion variants provide representative baselines ranging from simple feature aggregation to structured cross-modal interaction, enabling comprehensive analysis of the proposed conflict-aware FiLM modulation in fixed-budget risk screening scenarios.

### 2.6. Prototype-Guided Structured Risk Space

After completing conflict-aware multimodal fusion, the model needs to further organize the sample representations in a structured manner to support subsequent risk ranking and screening decisions. To this end, RiMS-FiLM introduces a prototype-guided mechanism to constrain the fused representations, enabling them to form an ordered distribution according to risk levels in the embedding space. Specifically, this module no longer relies on implicitly learned class boundaries in traditional classification models, but instead guides sample representations through a set of risk prototypes, enabling samples with similar risk levels to cluster together in the space while maintaining separation from other risk levels. In this way, the relative positions between samples can directly reflect their risk levels, providing a stable basis for subsequent risk scoring and prioritization.

#### 2.6.1. Risk Prototypes for Operational Stratification

Let $\mu_{k}\in R^{d}$ denote the learnable prototype vector associated with priority class k∈{L, M,H}. Each prototype represents the reference center of its corresponding class in the embedding space.

For a fused representation $z_{i}$, the squared Euclidean distance to prototype $\mu_{k}$ is computed as

(12) $$d_{ik}={{\left\|z_{i}-u_{k}\right\|}_{2}}^{2}$$

These distances characterize the relative proximity between each sample and the predefined risk centers.

#### 2.6.2. Geometry-Aware Risk Organization

To enable sample representations to support priority prediction, the model imposes prototype-based structural constraints in the embedding space. Specifically, for a sample representation $z_{i}$ with label $y_{i}=\{L,M,H\}$, training encourages $z_{i}$ to approach its target prototype $\mu_{y_{i}}$ while maintaining a margin from competing prototypes, thereby forming a class-structured distribution with explicit separation from non-target classes.

The relative distances between a sample and the three prototypes therefore characterize class-relative proximity in the embedding space. The prototype objective does not impose a separate ordinal distance constraint among the low-, medium-, and high-priority prototypes; rather, it provides class-structured regularization aligned with the three operational priority labels. This avoids post hoc clustering while keeping representation learning coupled to the downstream prediction objective.

#### 2.6.3. Prototype-Informed Risk Representation

The prototype module regularizes the fused representation rather than defining a separate ranking score. Distances to the low-, medium-, and high-priority prototypes provide structural supervision during training and encourage samples to occupy class-structured regions in the embedding space.

The fixed-budget ranking score itself is defined by the prediction head in Section 2.7.1; prototype geometry is analyzed only as a representation-space diagnostic in Section 3.5.

### 2.7. Training Objective and Optimization

The proposed RiMS-FiLM framework is trained end-to-end by jointly optimizing priori-ty-level classification and prototype-based representation regularization. Let $z_{i}$ denote the layer-normalized fused multimodal embedding obtained from Section 2.4 and Section 2.5, and let $y_{i}\in \{L,M,H\}$ denote the corresponding operational priority label.

#### 2.7.1. Risk Prediction Objective

The classifier first maps the fused embedding $z_{i}$ to base logits $l_{i}$. After the conflict-based high-priority logit refinement in Equation (8), the final logit vector is denoted by ${\tilde{l}}_{i}$, and the predicted class-probability vector is

(13) $$q_{i}=softmax({\tilde{l}}_{i})$$

where $q_{i}=[q_{i,L},q_{i,M},q_{i,H}]$ represents the predicted probabilities for the low-, medium-, and high-priority classes, respectively.

The priority-class prediction loss is the standard cross-entropy loss:

(14) $$L_{risk}=-\frac{1}{N}\sum_{i=1}^{N}logq_{i,y_{i}}$$

Although the training supervision is classification-based, fixed-budget screening is evaluated by ranking samples with a continuous score derived from the model probabilities.

In all reported fixed-budget experiments, the screening score is defined uniquely as $s_{i}=q_{i,H}$, and samples are ranked in descending order of this high-priority probability. No expected-priority score is used for the reported ranking metrics.

#### 2.7.2. Prototype Structure Learning Objective

To impose the class-structured constraint described in Section 2.6, the learnable prototypes $\mu_{k}$ are optimized with a margin-based regularization term. Using the squared distances $d_{ik}$ defined in Equation (12), the positive distance and the nearest non-target distance for sample i are defined as

(15) $$d_{i}^{+}={\left\|z_{i}-\mu_{y_{i}}\right\|}_{2}^{2},d_{i}^{-}=\min_{k\neq y_{i}}{\left\|z_{i}-\mu_{k}\right\|}_{2}^{2}$$

where $d_{i}^{+}$ is the squared distance to the target prototype and $d_{i}^{-}$ is the smallest squared distance to any non-target prototype. The prototype loss used in all reported experiments is

(16) $$L_{proto}=\frac{1}{N}\sum_{i=1}^{N}\max\left(0,\;d_{i}^{+}+m-d_{i}^{-}\right),\;m=0.2$$

The loss is zero when the nearest non-target prototype is at least the margin m farther than the target prototype; otherwise, a hinge penalty is applied. Thus, this term explicitly encourages proximity to the target prototype together with a margin from the nearest competing prototype.

The prototype term acts as representation regularization and does not define a separate screening score; fixed-budget ranking continues to use the high-priority probability defined in Section 2.7.1.

#### 2.7.3. Overall Objective and Optimization

The final training objective combines priority-level classification and prototype regularization:

(17) $$L=L_{risk}+{\lambda_{proto}L}_{proto}$$

where $\lambda_{proto}=0.05$ controls the contribution of the prototype regularization term. No separate conflict-specific auxiliary loss is used; the conflict head is optimized through L_risk_ because its output directly modifies the final logits in Equation (8).

All parameters of the visual encoder, physical encoder, fusion module, prototype vectors, and prediction head are jointly optimized using AdamW. This unified optimization ensures that multimodal representation learning, conflict-aware fusion, prototype regularization, and screening-oriented prediction are optimized within the same end-to-end model of high-priority sample prioritization under fixed inspection budgets.

## 3. Results and Discussion

### 3.1. Experimental Setup and Baseline Methods

#### 3.1.1. Implementation Details

All experiments are implemented using the PyTorch (Python 3.12) framework. The visual encoder adopts the Swin-T architecture (swin_tiny_patch4_window7_224) pretrained on ImageNet and fine-tuned on the GrainSet-Maize dataset. The physical encoder, fusion modules, prototype parameters, and prediction head are trained from scratch.

Models are optimized using the AdamW optimizer with an initial learning rate of 2 × 10^−4^ and a weight decay of 1 × 10^−2^. Training is conducted for up to 30 epochs with a batch size of 32. Early stopping is applied based on validation Macro-F1 with a patience of 7 epochs to prevent overfitting. Automatic mixed-precision training is enabled to improve computational efficiency. The principal comparisons and ablation experiments were repeated with five fixed random seeds (42, 123, 2024, 2025, and 2026); quantitative tables report mean ± standard deviation. For the CCL-SC baseline, five cross-entropy-only warm-up epochs preceded the contrastive stage, and the same validation Macro-F1/patience principle was applied after contrastive training began.

The hidden dimensions of the physical encoder and fusion layers are both set to 256, and cross-attention fusion employs eight attention heads. The prototype structure regularization weight is fixed at λ=0.05, while conflict-aware modulation is activated with a scaling factor of 1.0.

All experiments are performed on an NVIDIA RTX 3090 GPU (NVIDIA Corporation, Santa Clara, CA, USA). Comparable backbone, optimizer, batch-size, and evaluation settings are used across methods where applicable, while method-specific training components required by individual baselines are retained.

#### 3.1.2. Baseline Screening Paradigms

To comprehensively evaluate the proposed RiMS-FiLM framework under fixed-budget screening scenarios, four representative screening paradigms are selected as comparative baselines.

(1) Two-stage KMeans screening (TSK)

This method applies unsupervised KMeans clustering on multimodal embeddings to partition maize samples into several latent risk groups [19,20]. High-risk clusters are subsequently prioritized for reinspection according to cluster-level risk statistics.

(2) Selective classification network (SCN)

SelectiveNet-style models jointly learn prediction and confidence estimation, allowing uncertain samples to be rejected [28]. In this study, confidence scores are adapted as screening priorities for fixed-budget reinspection.

(3) Training-dynamics screening (TDS)

This approach leverages prediction stability and learning difficulty across training epochs as indicators of risk, ranking samples with unstable training dynamics as high-priority candidates [29].

(4) Confidence-aware contrastive screening (CCS)

We implement the confidence-aware contrastive learning for selective classification (CCL-SC) formulation [30] and adapt its output to fixed-budget screening; this adapted baseline is denoted CCS in the tables and figures for brevity.

All baselines are implemented under the same multimodal encoder backbone where applicable and converted into ranking-based screening strategies for fair comparison under identical budget constraints.

#### 3.1.3. Complementary Classification Performance Metrics

In addition to the budget-aware screening metrics defined in Section 2.2.3, conventional classification metrics are reported to provide complementary evaluation of risk prediction performance.

Specifically, Macro-F1 and Micro-F1 scores are computed across the three risk levels. Macro-F1 equally weights each risk category and is particularly suitable for assessing robustness under class imbalance, while Micro-F1 reflects overall prediction accuracy dominated by majority classes. These metrics serve as auxiliary indicators and do not directly represent operational screening effectiveness, which is primarily assessed through ${Recall}_{H}@B$, ${Missed}_{H}@B$, and AURC.

Specifically, Macro-F1 and Micro-F1 scores are computed across the three priority levels. Macro-F1 equally weights each priority category and is particularly suitable for assessing robustness under class imbalance, while Micro-F1 reflects overall predictive accuracy. These metrics serve as complementary indicators and do not directly represent operational screening effectiveness, which is primarily assessed through ${Recall}_{high}@B$, ${Missed}_{high}@B$, and $p{AURC}_{0.30}$.

### 3.2. Fixed-Budget Screening Performance Comparison

#### 3.2.1. High-Risk Prioritization Under Fixed Budgets

We first compare RiMS-FiLM against four representative screening paradigms under the fixed-budget setting defined in Section 2.2. Table 4 summarizes high-priority recall and missed-high rates at representative inspection budgets B.

Across five random seeds, RiMS-FiLM retrieves 23.75% of high-priority kernels at a 5% budget and 47.50% at a 10% budget, reaching the theoretical maximum at both operating points.

At a 20% re-inspection budget, RiMS-FiLM achieves a mean high-priority recall of 0.9465 ± 0.0021, compared with 0.9420 ± 0.0023 for CCS, and reaches 0.9995 ± 0.0007 at a 30% budget.

These results indicate that RiMS-FiLM is able to front-load high-priority kernels within limited inspection budgets, a property relevant to real-world grain storage quality control.

#### 3.2.2. Risk–Coverage Behavior and Screening Efficiency

Figure 4 summarizes the five-seed mean missed-high rate at representative inspection budgets. RiMS-FiLM remains near the lower envelope of missed-high rates over the operational range, consistent with its lowest mean $p{AURC}_{0.30}$ among the screening baselines.

Figure 5 reports the corresponding five-seed mean high-priority recall at the same representative budgets, with error bars showing the standard deviation across seeds. RiMS-FiLM reaches the theoretical ceiling at 5% and 10% budgets and remains close to the attainable maximum at 20% and 30%.

$p{AURC}_{0.30}$ (missed-high) represents cumulative missed detection over the opera-tional budget range B∈[0,0.30]; a lower value indicates faster high-priority sample discovery. As presented in Table 5, RiMS-FiLM achieved the lowest mean $p{AURC}_{0.30}$ among the compared screening baselines (0.1056 ± 0.0001), compared with 0.1059 ± 0.0002 for CCS, and substantially lower values than SCN and TDS. Under the same test-set prevalence, the theoretical continuous lower bound is approximately 0.1053, so the observed RiMS-FiLM value is close to the attainable lower bound over this budget range.

#### 3.2.3. Joint Analysis with Classification Robustness

Beyond operational screening efficiency, Table 5 also reports Macro-F1 and Micro-F1 scores to evaluate the robustness of risk prediction under class imbalance.

RiMS-FiLM achieves the highest Macro-F1 among all methods, indicating balanced recognition capability across low-, medium-, and high-risk levels. This improvement is particularly important in grain storage scenarios where high-risk samples constitute a minority yet carry the greatest operational importance. Meanwhile, Micro-F1 remains competitive, reflecting strong overall predictive accuracy.

The combination of the lowest mean $p{AURC}_{0.30}$ among the screening baselines and the highest Macro-F1 indicates that RiMS-FiLM provides both efficient early retrieval and balanced three-class prediction. These quantitative results are consistent with the intended representation design, while the diagnostic visualizations below are interpreted descriptively rather than as stand-alone causal proof of the internal mechanism.

As an additional grouped robustness evaluation, RiMS-FiLM retained its performance when identical (DU_grain, weight) groups were prevented from crossing partitions: over five seeds, Macro-F1 was 0.9940 ± 0.0014 and Recall@20% was 0.9485 ± 0.0010. Under the same grouped protocol, CCS achieved 0.9850 ± 0.0025 and 0.9428 ± 0.0030, respectively, while the vision-only ablation achieved 0.9849 ± 0.0023 and 0.9433 ± 0.0029. This result indicates that the main finding is retained under the more conservative group-disjoint split.

#### 3.2.4. Discussion: Implications for Operational Grain Risk Management

The findings demonstrate that, under a fixed re-inspection budget, screening effectiveness depends more on how quickly the model ranks high-risk samples near the top than on overall classification accuracy. In addition, there may be substantial differences in the actual value of the different types of methods when used for actual screenings. This is particularly evident within the lower budget range; therefore, even small differences in rank position can produce significant missed opportunities for high-risk kernels.

Similarly, predictive assessment tools based on either confidence-based or dynamic training do not actually characterize risk by prediction uncertainty. While these methods may have some advantages in an overall abstention-based behavior model, the ranking structures of these methods are typically not stable, especially when budgets are limited when the highest-risk samples will be primarily distributed throughout the midrange and low-end of the sample rankings while limiting the effectiveness of earlier screening methods. Additionally, representative-space detection strategies will provide smoother risk coverage curves; however, misalignment of screening priorities can still occur, at the borderline between medium-and high-risk samples.

RiMS-FiLM does not utilize post hoc adjustments to the confidence in the ranking like these methods but rather shapes the ranking space through structured representation learning. To avoid relying too heavily on one modality, the combination of conflicting multimodal feature fusion mechanisms allows the model to modify feature responses based on the inconsistency of both the visual and physical domains. Additionally, the prototype-based risk structure constraints increase the separation of different risk types within the embedding space thereby allowing higher risk instances to be located more stably in front of the ranking system. The mechanism of creating structural representations at the representation level and then using that structural representation to reflect a ranking at the decision level provides for consistent behavior in the screening process regardless of the budget.

With respect to being able to manage storage from a practical standpoint there are very direct implications for operation. When storing or moving large amounts of grain, the limiting factor will almost always be the resources used to re-inspect product. Thus, the key area for making decisions will not be ‘Is my decision correct?’, but rather ‘Which samples have the highest risk and should be inspected sooner than later?’. So even if a screening type of model can just raise the rank of those high-risk samples reliably, there may be great economic savings and a significant reduction in risk as it relates to quality due to practical applications.

Further studies should evolve from a “prediction-based” model development paradigm to a “decision-based” model development paradigm, as discussed above. Using classification confidence or error minimizer objectives in isolation does not demonstrate the actual utility of the models when resources are limited. Therefore, developing the risk structure in the representation and aligning a rank objective with the risk would appear to be the best way to maximize utility in these types of problems. This study’s performance of RiMS-FiLM accordingly supports the above argument through evidential validation of its potential.

### 3.3. Mechanistic Comparison with CCS

Because CCS is the strongest screening baseline in Table 4 and Table 5, we further compare it with RiMS-FiLM using the final five-seed results. This comparison focuses on reproducible classification and budget-aware screening metrics rather than inferring internal mechanisms from a single confusion matrix or discrete score plot.

#### 3.3.1. Five-Seed Classification Comparison

Figure 6 compares Macro-F1 and Micro-F1 across five seeds. RiMS-FiLM achieves 0.9917 ± 0.0027 Macro-F1 and 0.9927 ± 0.0024 Micro-F1, compared with 0.9858 ± 0.0018 and 0.9877 ± 0.0015 for CCS, respectively.

The advantage is therefore observed in both balanced class-wise performance and overall predictive accuracy, while the standard deviations remain small for both methods.

These five-seed results provide the quantitative basis for comparing the two methods; they replace reliance on the earlier single-run confusion-matrix interpretation.

#### 3.3.2. Fixed-Budget Ranking Comparison

Figure 7 compares high-priority recall at the four reported re-inspection budgets. Both methods reach the theoretical ceilings at 5% and 10%. At a 20% budget, RiMS-FiLM achieves 0.9465 ± 0.0021 compared with 0.9420 ± 0.0023 for CCS; at 30%, the two methods are both close to complete high-priority coverage.

Together with the $p{AURC}_{0.30}$ values in Table 5 (0.1056 ± 0.0001 for RiMS-FiLM and 0.1059 ± 0.0002 for CCS), the results show a small but consistent advantage for RiMS-FiLM in early high-priority retrieval under the primary stratified protocol.

### 3.4. Effectiveness of Multimodal Fusion Strategies

To analyze the impact of different multimodal interaction mechanisms on risk representation and screening stability, this paper compares the FiLM fusion method with three typical strategies: feature concatenation, gated weighted fusion, and attention-based cross-modal interaction. Under the premise of maintaining consistent encoder structure and training settings, the above methods are all embedded into the unified risk screening framework for evaluation.

In addition to the five-seed metrics, Figure 8 provides a single-run diagnostic view of boundary-sensitive classification errors for the fusion variants. This visualization is descriptive only; quantitative conclusions are based on the five-seed summaries in Table 6. The integrated screening metric is $p{AURC}_{0.30}$ as defined in Section 2.2.3.

Figure 8 presents the performance of different fusion strategies on three critical error types from a screening decision perspective, including high-risk samples misclassified as low risk, classification deviation of medium-risk samples, and non-high-risk samples misclassified as high risk.

On the most critical severe miss metric, the differences are most pronounced in this diagnostic run. FiLM controls this error rate at 0.005, while Concat, Gated-Sum, and Cross-Attention reach 0.028, 0.010, and 0.014, respectively. Notably, Concat’s error rate exceeds FiLM’s by more than five times, indicating that in the absence of effective cross-modal modulation, high-risk features are more likely to be attenuated during fusion, thereby being incorrectly suppressed to the lowest priority region.

Based on the medium-risk error metric, FiLM has the lowest risk score at 0.001. The other methods were at risk scores of 0.015 for Concat, 0.006 for Gated-Sum, and 0.007 for Cross-Attention. The effect of this error type is limited on overall accuracy, but it could create an issue with continuity in the ranking structure based on the cumulative effect in terms of risk stratification.

In this single-run diagnostic, FiLM shows lower boundary-sensitive error rates than the alternative fusion variants. These patterns provide qualitative context for Table 6 but are not used to claim statistical dominance on every screening metric.

The five-seed results in Table 6 provide the primary evidence: RiMS-FiLM attains the highest mean Macro-F1 and Micro-F1 among the fusion variants while maintaining near-ceiling fixed-budget screening performance.

Table 6 compares different multimodal fusion strategies from two perspectives: overall screening performance and classification stability. Overall, the differences between methods in $p{AURC}_{0.30}$ are relatively close, indicating that at the global risk-coverage curve level, different fusion methods are all able to form relatively similar ranking trends.

At the 20% re-inspection budget, all strong fusion variants remain close to the theoretical ceiling, with mean Recall@20% values ranging from 0.9455 to 0.9475; RiMS-FiLM achieves 0.9465 ± 0.0021.

RiMS-FiLM achieves the highest mean Macro-F1 (0.9917 ± 0.0027) and Micro-F1 (0.9927 ± 0.0024) among the fusion variants, while maintaining near-ceiling fixed-budget screening performance.

Accordingly, the boundary-error visualization should be read as a descriptive complement to the five-seed results. The principal quantitative conclusion is that FiLM provides the strongest mean classification performance among the tested fusion strategies without sacrificing screening efficiency near the operational ceiling.

### 3.5. Ablation Study and Structural Interpretation of RiMS-FiLM

To further analyze the specific roles of each core module of RiMS-FiLM in risk screening, this paper conducts ablation experiments by progressively removing key components, including physical feature inputs, prototype-guided risk structure, and conflict-aware modulation mechanism. The corresponding quantitative results are shown in Table 7, while Figure 9 and Figure 10 provide single-run diagnostic visualizations of the changes in representation structure and screening behavior under different component removal conditions. Under the premise of maintaining consistent training settings and the remaining model structure, the above ablation experiments aim to evaluate the contribution of each module to risk ranking stability and screening effectiveness from two levels: overall performance and structural performance. Compared with analysis methods that rely solely on metric changes, combining the comparison of embedding space distribution and risk ranking behavior can more intuitively reveal the impact of different components on the model’s internal risk organization mechanism.

#### 3.5.1. Impact of Prototype-Guided Risk Structure

As shown in Table 7, removing the prototype-guided structure produces a small decrease in mean Macro-F1, from 0.9917 ± 0.0027 to 0.9908 ± 0.0030, while the fixed-budget ranking metrics remain close to saturation. The five-seed results therefore support the prototype term primarily as a representation-structure regularizer rather than as a component that must improve every individual screening metric.

Figure 9 provides a descriptive view of how the prototype regularizer changes the geometry of the learned representation. In the with-prototype condition, the Top-B samples show a more concentrated distance pattern relative to the class reference centers, and the margin distribution shifts toward larger separation values compared with the no-prototype condition. This is consistent with the explicit margin constraint in Equation (16).

Figure 9c shows that the margin distribution for the selected Top-B samples is generally shifted toward larger values when prototype regularization is used. Figure 9d further illustrates the margin behavior of missed high-priority samples. These plots are descriptive diagnostics of representation geometry and are not interpreted as independent causal proof of screening performance.

Combining Table 7 and Figure 9, it can be seen that the role of the prototype-guided structure in RiMS-FiLM is mainly reflected in two aspects: on one hand, it provides explicit risk-level reference for multimodal representations, enabling samples to form a more ordered organization in the embedding space; on the other hand, it enlarges the geometric margin near the screening boundary, thereby improving the stability of high-risk samples being preferentially selected under limited budget conditions. Therefore, this module provides an explicit structural regularizer for the multimodal representation. These representation-space visualizations are descriptive and are not used as causal proof of the mechanism.

#### 3.5.2. Effect of Multimodal Physical Feature Integration

We further analyze the role of physical features in multimodal fusion. Removing the physical branch produces the clearest ablation effect: Table 7 shows that Macro-F1 decreases from 0.9917 ± 0.0027 to 0.9859 ± 0.0014, Recall@20% decreases from 0.9465 ± 0.0021 to 0.9420 ± 0.0026, and $p{AURC}_{0.30}$ increases from 0.1056 ± 0.0001 to 0.1060 ± 0.0001. This indicates that physical attributes provide complementary information beyond visual appearance.

This transition illustrates that using only the visual modality cannot provide all the characteristics necessary for assessing the operational re-inspection priority of maize kernels; some high-risk samples may be visually indistinguishable, but have previously identified weight/size anomalies. Information about the sample will be unusable or inadequate without a physical representation, which leads to bias in the model’s determination of sample risk.

With regard to the screening process, this bias manifests as an overall backward shift in the ranking positions of high-priority abnormal samples. Due to the lack of supporting visual evidence, the model is more likely to classify a sample as “visually normal” and place it in the low-risk area even if the sample warrants a higher re-inspection priority. This has important implications for re-inspection under limited budgets, where ranking position directly impacts whether a sample will be included in the re-inspection set.

In general, physical features complement visual information and help stabilize risk judgment when integrating different modalities. Removing physical features from the model reduces the model’s ability to identify potentially high-priority abnormal samples due to lack of information, ultimately resulting in lower overall screening effectiveness.

#### 3.5.3. Contribution of Conflict-Aware Modulation

Finally, we analyze the role of the conflict-aware modulation mechanism. Across five seeds, removing this mechanism changes the mean Macro-F1 from 0.9917 ± 0.0027 to 0.9910 ± 0.0027, Recall@20% from 0.9465 ± 0.0021 to 0.9463 ± 0.0027, and $p{AURC}_{0.30}$ from 0.1056 ± 0.0001 to 0.1057 ± 0.0001. The numerical effect is modest; Figure 10 is therefore used to describe how the conflict score behaves rather than to claim a large independent performance gain.

Figure 10 shows the conflict-score distributions for selected and missed high-priority samples in a single diagnostic run, with and without the conflict-aware branch. The distributions differ across these groups, indicating that the explicit discrepancy signal participates in the model’s decision pathway. However, the conflict score is not monotonic with prediction error and should not be interpreted as a calibrated uncertainty measure.

The comparison without the conflict branch further shows that the distributional pattern changes when the explicit discrepancy-based logit adjustment is removed. Because this visualization is single-run and descriptive, it is used only to illustrate how the conflict signal behaves, while the five-seed ablation values in Table 7 provide the quantitative evidence for the component’s contribution.

Accordingly, the conflict-aware branch is best interpreted as an additional cross-modal discrepancy adjustment rather than a stand-alone uncertainty detector. Its mean quantitative effect is modest, but the full model retains slightly higher Macro-F1 and comparable near-ceiling screening performance across the five seeds.

## 4. Conclusions

Under a fixed re-inspection budget, operational grain screening is not only a classification task but also a prioritization problem. RiMS-FiLM combines non-destructive RGB appearance with lightweight physical metadata to rank maize kernels for follow-up verification. Across five random seeds on the primary stratified split, the model achieved Recall@20% = 0.9465 ± 0.0021 and Macro-F1 = 0.9917 ± 0.0027. Because the risk tiers are operational labels derived from defect categories rather than laboratory-validated toxicological endpoints, these results should be interpreted as re-inspection-priority performance for quality control.

Ablation and diagnostic analyses are consistent with complementary roles for the three main components: prototype-guided learning provides class-structured regularization, physical metadata supplement visual cues, and FiLM-based conditional modulation with the discrepancy branch provides sample-dependent cross-modal adjustment. Together, these components support prioritization near the re-inspection cutoff, where ranking errors have the greatest operational consequence.

Overall, the study demonstrates the potential of low-cost multimodal sensing and machine learning for non-destructive grain quality screening under resource constraints. The present study is limited to one public dataset and does not include independent external validation, real warehouse testing, laboratory-confirmed endpoints, or a dedicated sensor-variability analysis. Future work should validate the framework against laboratory-measured physicochemical or safety endpoints, evaluate external batches and distribution shifts, and investigate dynamic inspection budgets, additional sensing modalities, and uncertainty-calibrated decision rules.

## Figures and Tables

**Figure 1:**
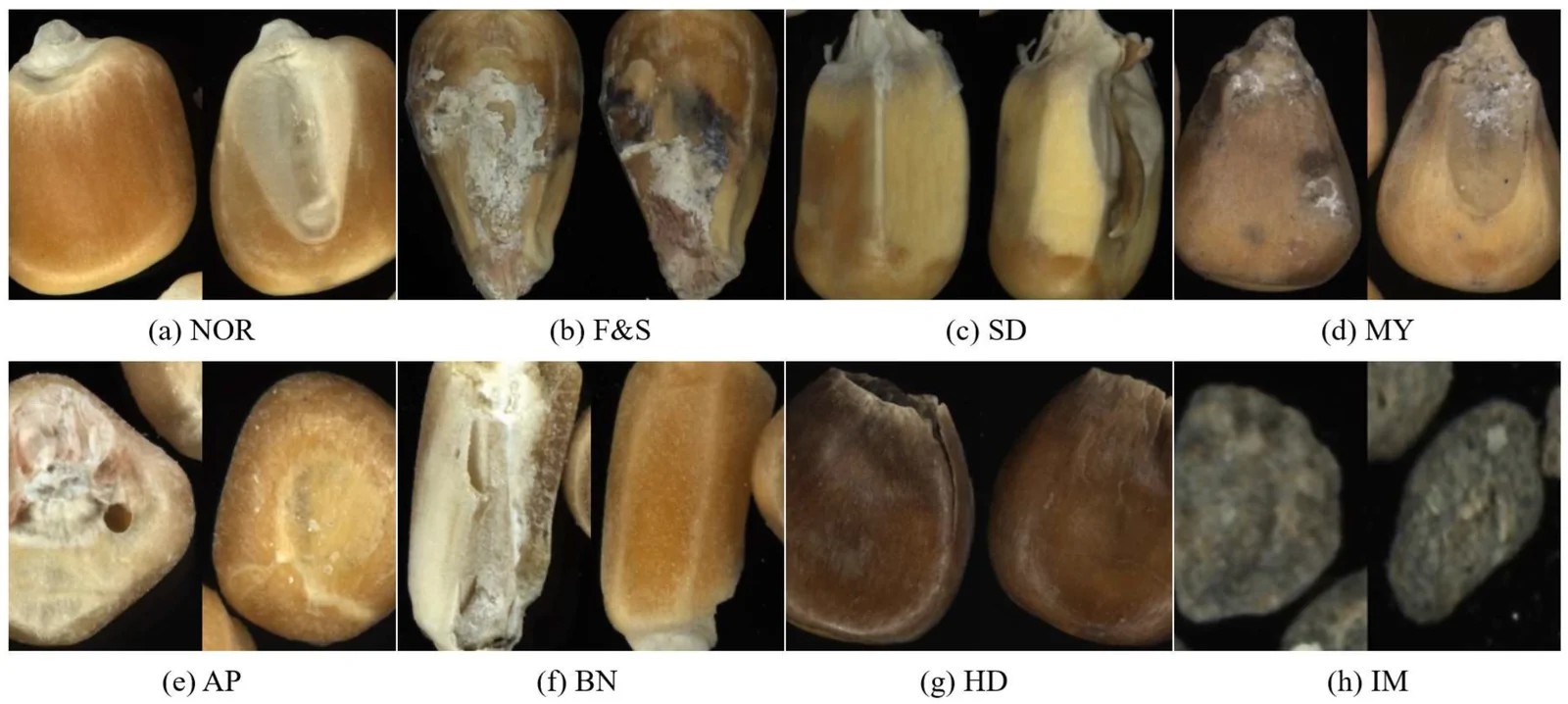
Example images of the eight maize quality categories. Source: GrainSet-Maize [23]. NOR, Normal; F&S, Fusarium & Shriveled; SD, Sprouted; MY, Moldy; AP, Attacked by Pests; BN, Broken; HD, Heat Damaged; IM, Impurities.

**Figure 2:**
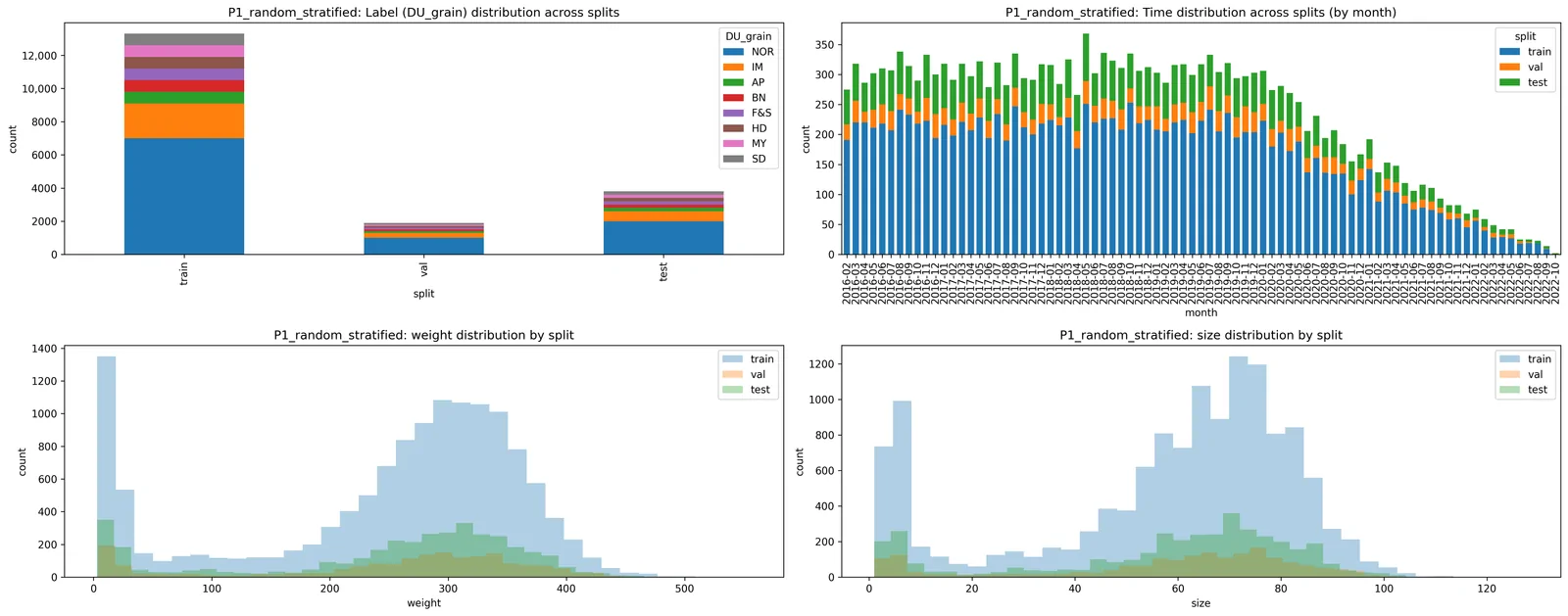
Statistical distribution comparison of labels, acquisition time, and physical attributes across data splits under the proposed risk-stratified protocol.

**Figure 3:**
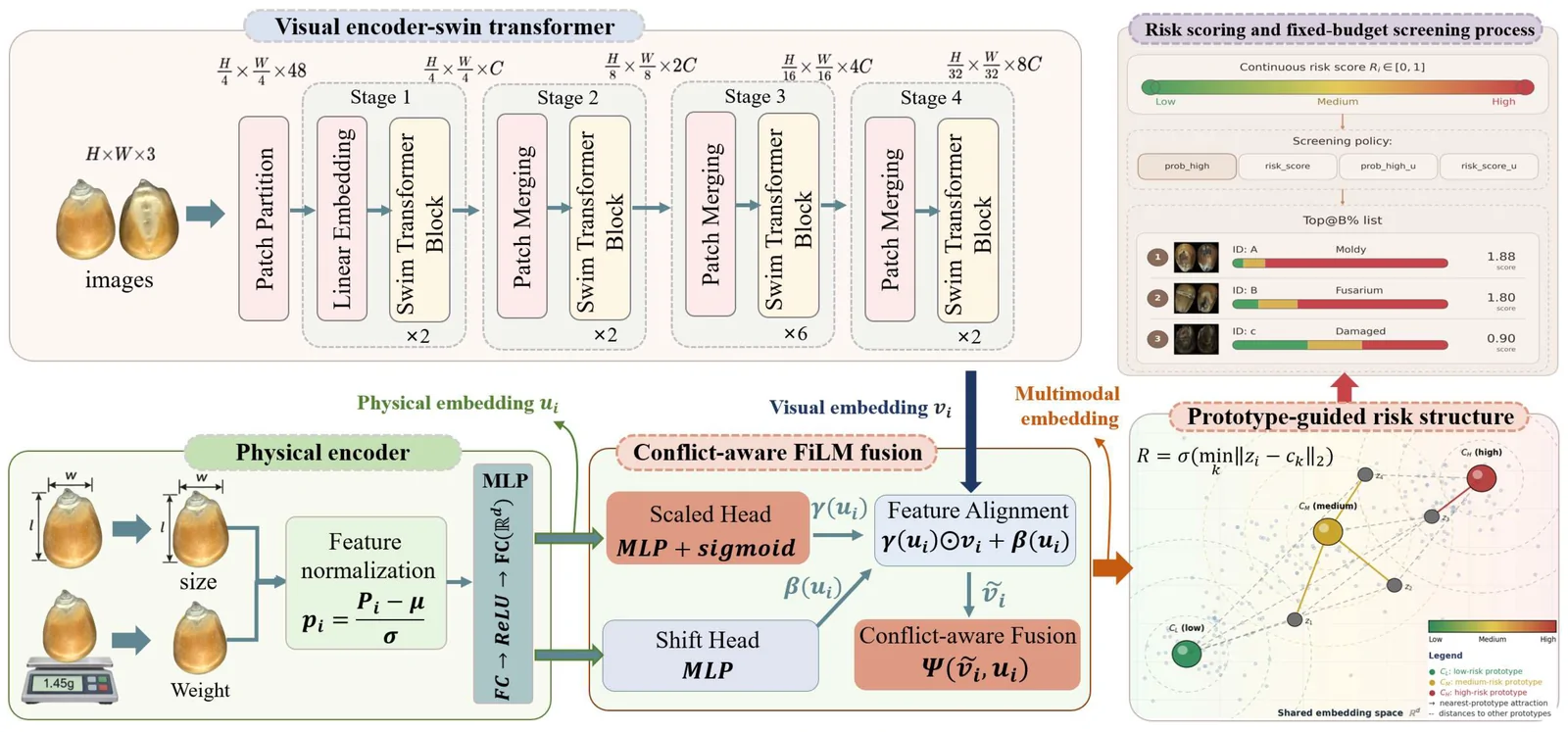
Overall architecture of the proposed RiMS-FiLM.

**Figure 4:**
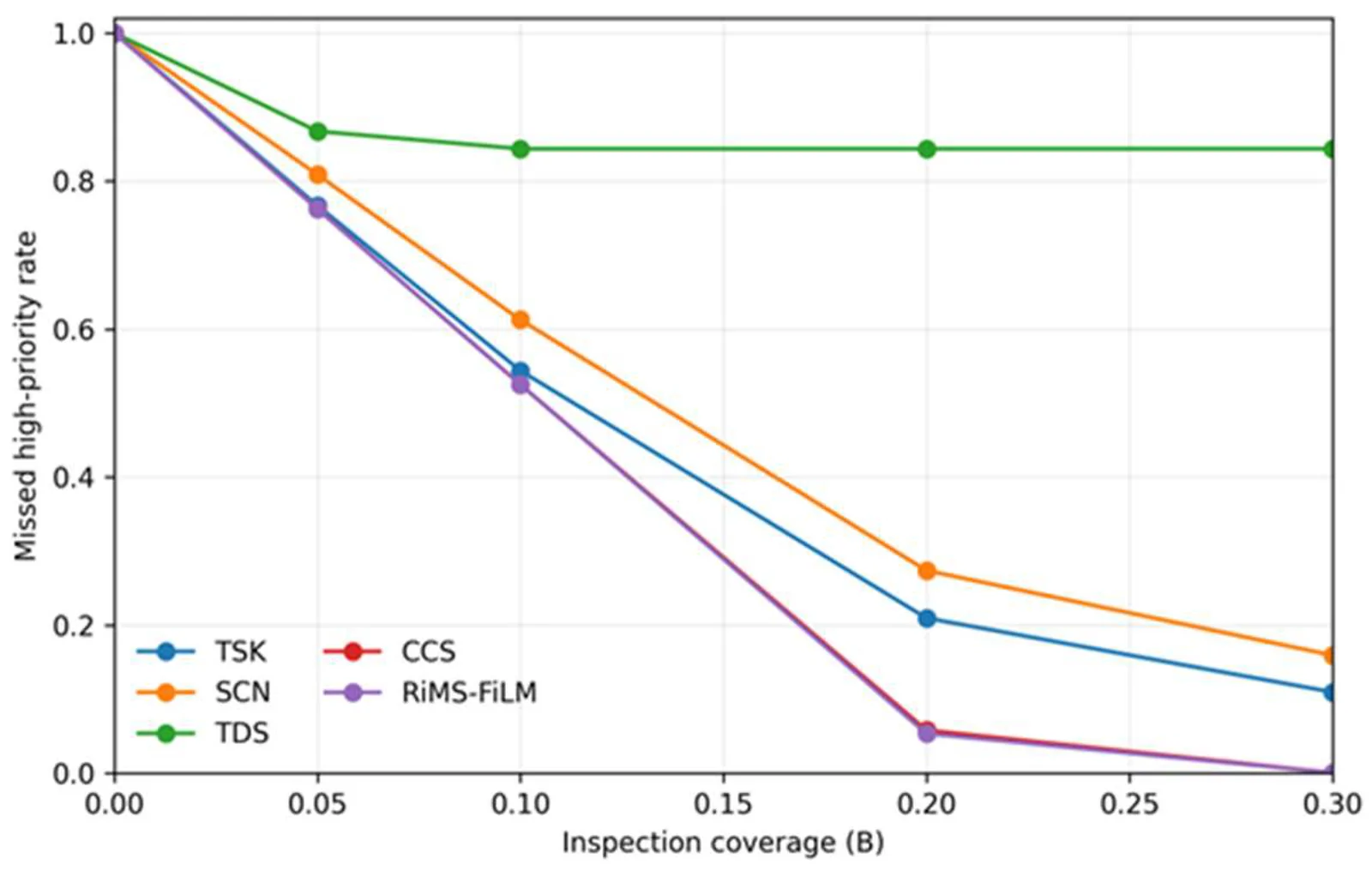
Five-seed mean missed-high rate at representative inspection budgets. Lines connect the re-ported budget points for visualization; $p{AURC}_{0.30}$ is computed from all attainable sample-level budget points.

**Figure 5:**
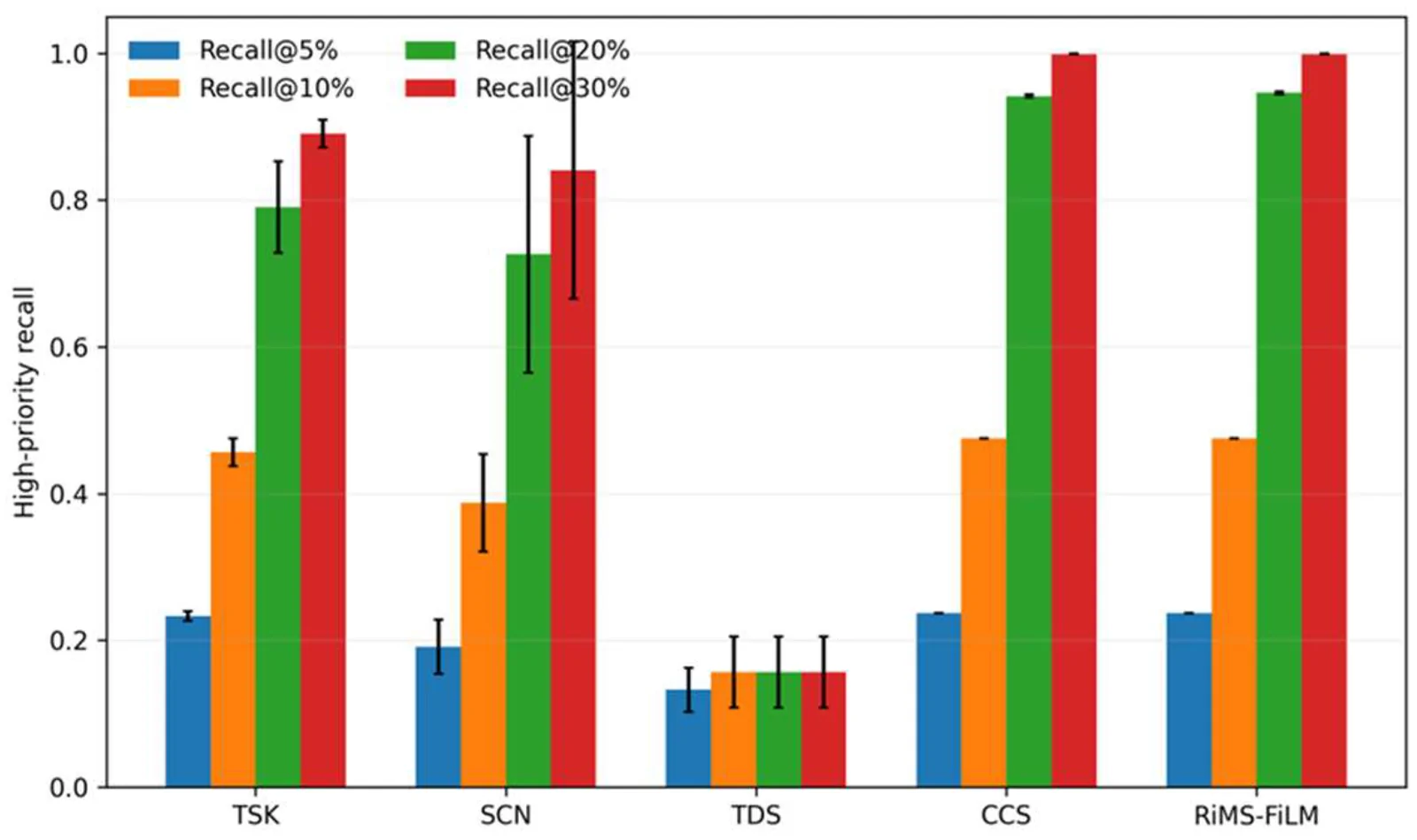
Five-seed mean high-priority recall at representative inspection budgets; error bars denote standard deviation across the five seeds.

**Figure 6:**
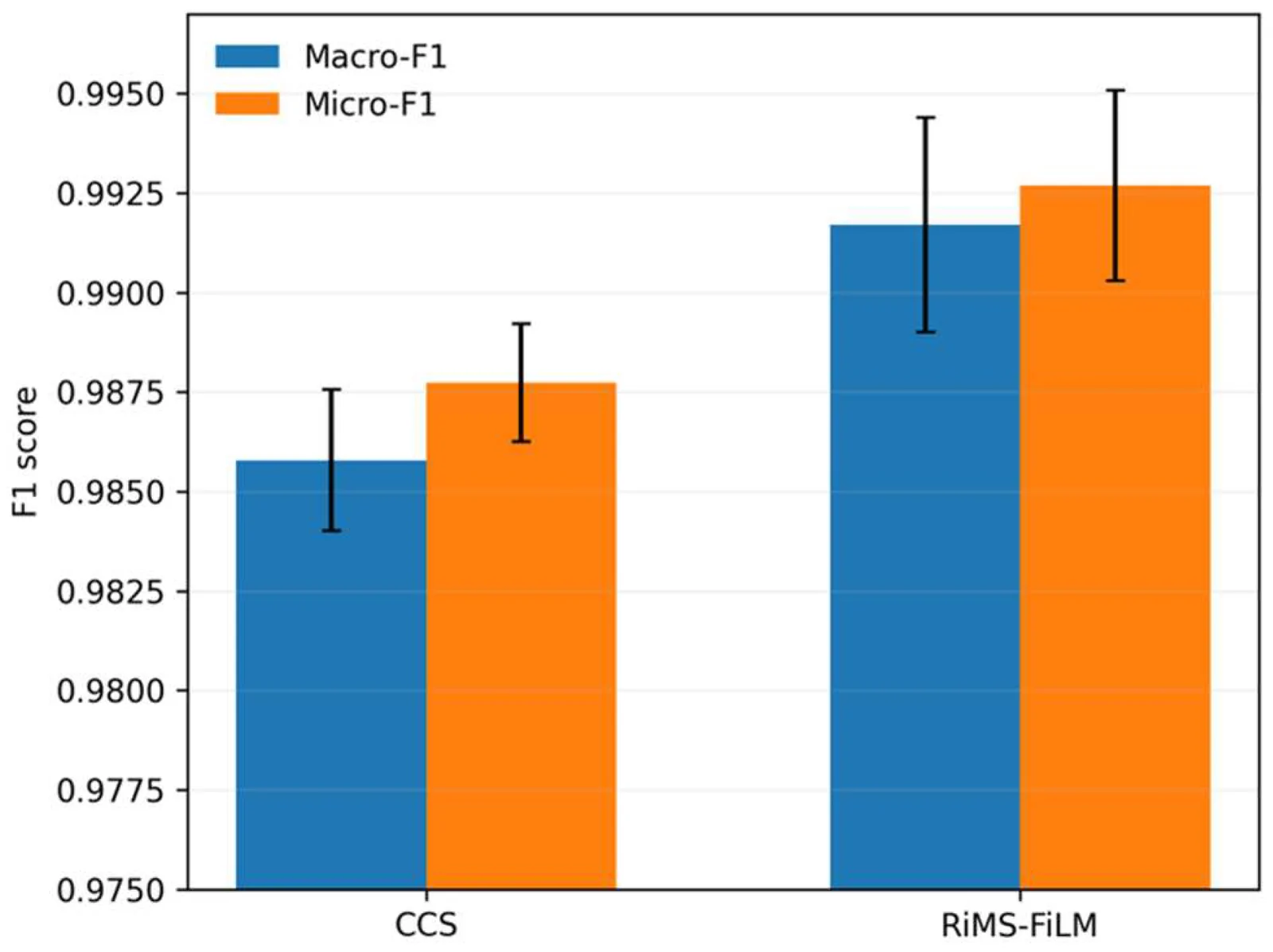
Five-seed Macro-F1 and Micro-F1 comparison between RiMS-FiLM and CCS; error bars denote standard deviation.

**Figure 7:**
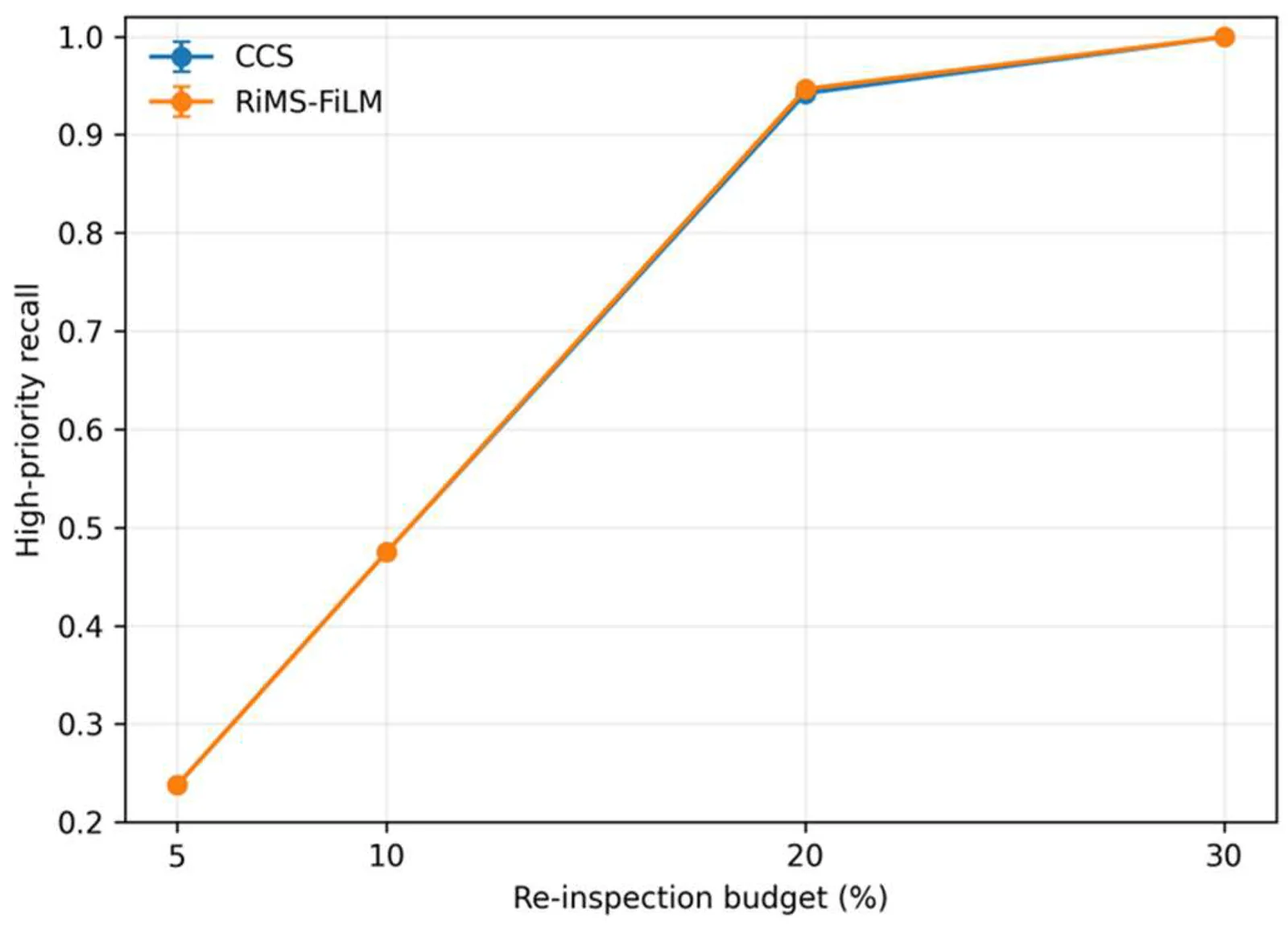
Five-seed high-priority recall of RiMS-FiLM and CCS at representative re-inspection budgets; error bars denote standard deviation.

**Figure 8:**
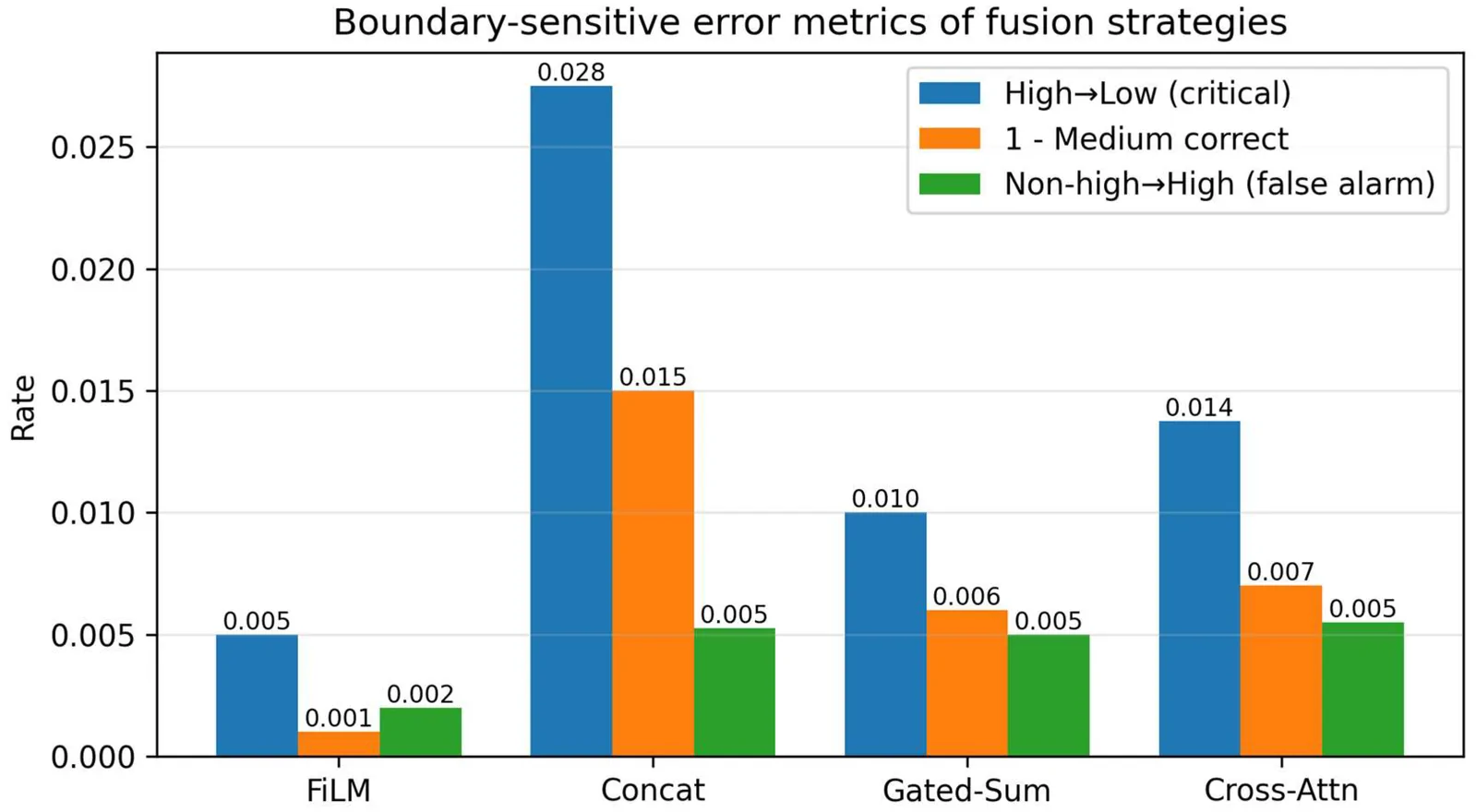
Boundary-sensitive error analysis of multimodal fusion strategies under fixed-budget screening.

**Figure 9:**
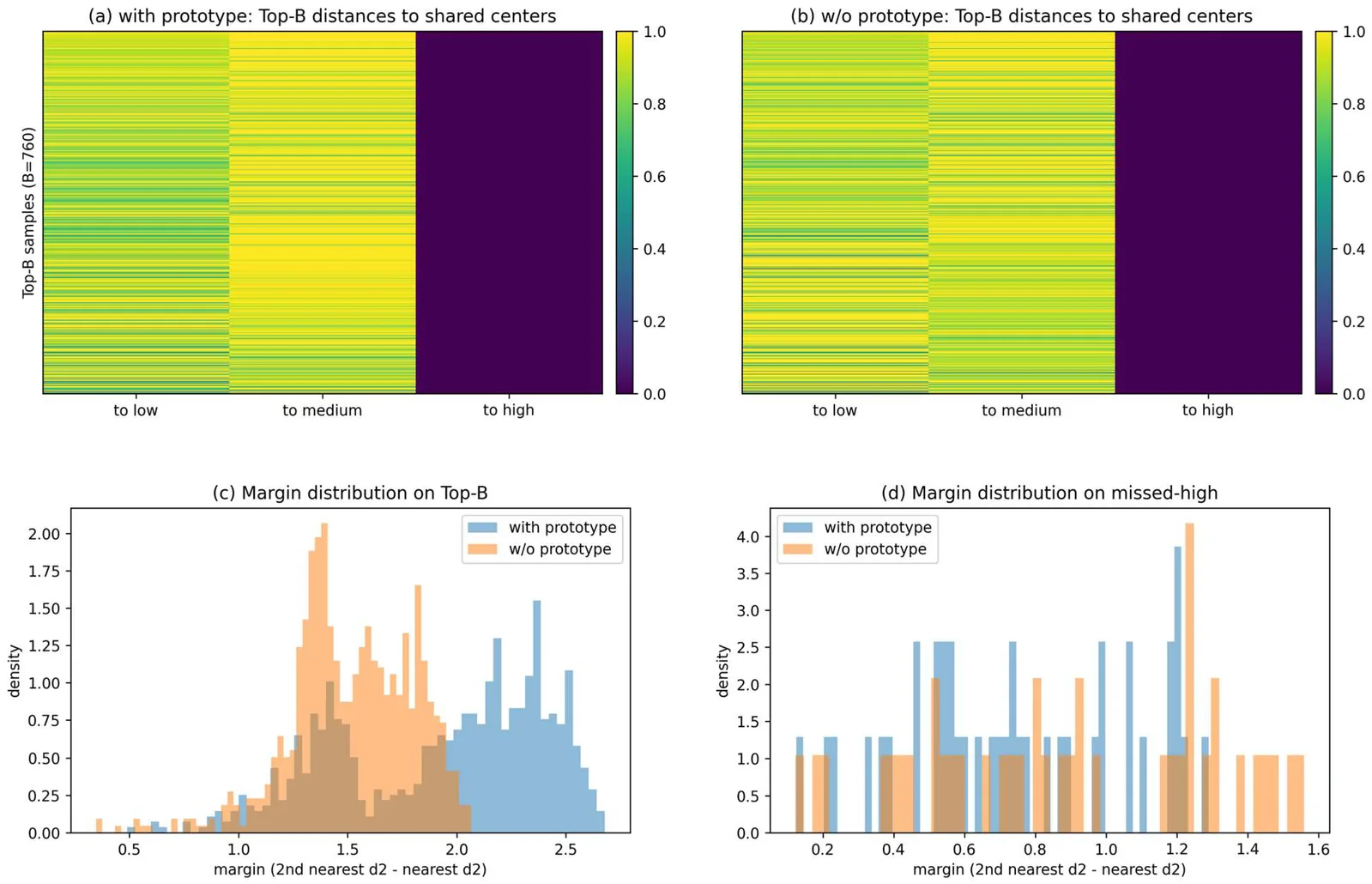
Effect of prototype-guided risk structure on geometric separation and margin distribution under fixed-budget screening.

**Figure 10:**
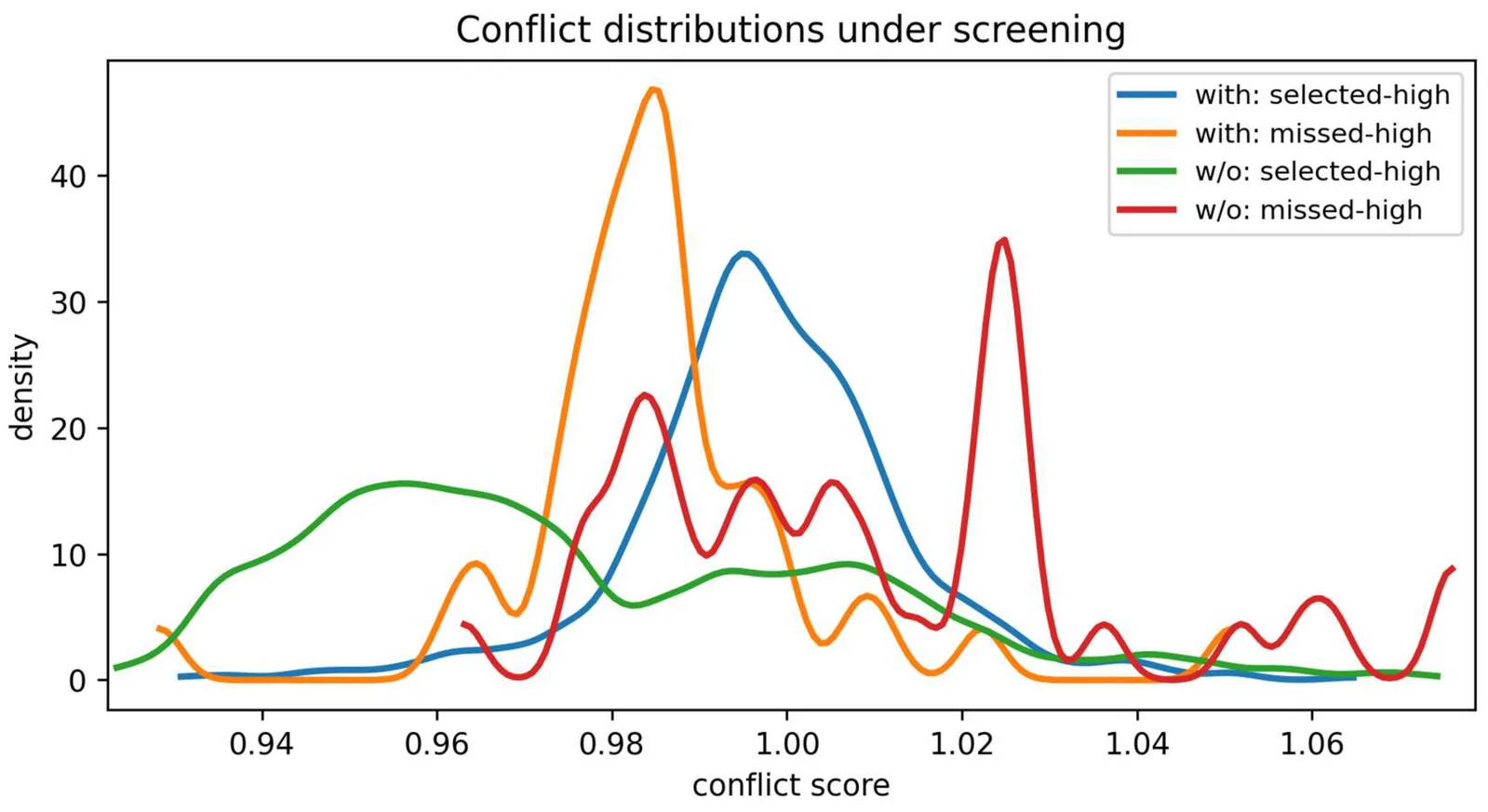
Impact of conflict-aware modulation on screening behavior and conflict-score distributions.

**Table 1:** Example of a structured metadata record associated with a single maize kernel sample in the GrainSet-Maize dataset.

Metadata Field	Example Value
ID	Grainset_maize_2020-06-04-11-14-29_0_p600s
species	maize
sub-species	popped corn
location	CN (China)
Time	9 November 2017
Size	45 (mm^2^)
DU_grain	BN (Broken)
Weight	134 (g; batch-associated)

**Table 2:** Official train–test distribution of the GrainSet-Maize subset across eight quality categories. Values are numbers of kernel images in the official dataset split.

	NOR	F&S	SD	MY	AP	BN	HD	IM
train	9000	900	900	900	900	900	900	2700
test	1000	100	100	100	100	100	100	300
total	10,000	1000	1000	1000	1000	1000	1000	3000

**Table 3:** Mapping between original defect categories and operational quality-risk tiers for maize kernel screening.

Defect Category	Operational Quality-Risk Tier
Normal (NOR)	Low
Broken (BN)	Medium
Sprouted (SD)	High
Moldy (MY)	High
Fusarium & Shriveled (F&S)	High
Attacked by pests (AP)	High
Heat-damaged (HD)	Medium
Impurities (IM)	Medium

**Table 4:** Fixed-budget screening performance (${Recall}_{high}@B$, ${Missed}_{high}@B$ at B=5%, 10%, 20%, 30%). Values are mean ± SD over five seeds.

Model	Recall5	Recall10	Recall20	Recall30	Missed5	Missed10	Missed20	Missed30
TSK	0.2330 ± 0.0068	0.4563 ± 0.0189	0.7905 ± 0.0625	0.8905 ± 0.0190	0.7670 ± 0.0068	0.5438 ± 0.0189	0.2095 ± 0.0625	0.1095 ± 0.0190
SCN	0.1913 ± 0.0368	0.3875 ± 0.0667	0.7263 ± 0.1614	0.8408 ± 0.1753	0.8088 ± 0.0368	0.6125 ± 0.0667	0.2738 ± 0.1614	0.1593 ± 0.1753
TDS	0.1328 ± 0.0299	0.1565 ± 0.0483	0.1565 ± 0.0483	0.1565 ± 0.0483	0.8673 ± 0.0299	0.8435 ± 0.0483	0.8435 ± 0.0483	0.8435 ± 0.0483
CCS	0.2375 ± 0.0000	0.4750 ± 0.0000	0.9420 ± 0.0023	0.9995 ± 0.0007	0.7625 ± 0.0000	0.5250 ± 0.0000	0.0580 ± 0.0023	0.0005 ± 0.0007
RiMS-FiLM	0.2375 ± 0.0000	0.4750 ± 0.0000	0.9465 ± 0.0021	0.9995 ± 0.0007	0.7625 ± 0.0000	0.5250 ± 0.0000	0.0535 ± 0.0021	0.0005 ± 0.0007

Because the test set contains 800 high-priority kernels among 3800 samples (21.05%), the theoretical maximum Recall@B values at B = 5%, 10%, 20%, and 30% are 0.2375, 0.4750, 0.9500, and 1.0000, respectively. RiMS-FiLM reaches the first two ceilings and remains close to the theoretical maximum at 20% and 30%.

**Table 5:** Joint evaluation of screening efficiency and classification robustness on the test set ($p{AURC}_{0.30}$, Macro-F1, and Micro-F1; mean ± SD over five seeds).

Model	$p{AURC}_{0.30}$ ↓	Macro-F1 ↑	Micro-F1 ↑
TSK	0.1302 ± 0.0097	0.8995 ± 0.0404	0.9145 ± 0.0345
SCN	0.1444 ± 0.0309	0.9570 ± 0.0324	0.9648 ± 0.0262
TDS	0.2580 ± 0.0124	0.9846 ± 0.0017	0.9868 ± 0.0013
CCS	0.1059 ± 0.0002	0.9858 ± 0.0018	0.9877 ± 0.0015
RiMS-FiLM	0.1056 ± 0.0001	0.9917 ± 0.0027	0.9927 ± 0.0024

**Table 6:** Performance comparison of multimodal fusion mechanisms in fixed-budget risk screening (mean ± SD over five seeds).

Model	$p{AURC}_{0.30}$ ↓	Recall5	Recall10	Recall20	Recall30	Macro-F1 ↑	Micro-F1 ↑
RiMS-Concat	0.1058 ± 0.0002	0.2375 ± 0.0000	0.4750 ± 0.0000	0.9470 ± 0.0019	0.9990 ± 0.0006	0.9897 ± 0.0034	0.9909 ± 0.0030
RiMS-GatedSum	0.1056 ± 0.0001	0.2375 ± 0.0000	0.4750 ± 0.0000	0.9455 ± 0.0031	1.0000 ± 0.0000	0.9900 ± 0.0039	0.9914 ± 0.0031
RiMS-CrossAttn	0.1056 ± 0.0002	0.2375 ± 0.0000	0.4748 ± 0.0006	0.9475 ± 0.0023	0.9993 ± 0.0007	0.9911 ± 0.0030	0.9922 ± 0.0025
RiMS-FiLM	0.1056 ± 0.0001	0.2375 ± 0.0000	0.4750 ± 0.0000	0.9465 ± 0.0021	0.9995 ± 0.0007	0.9917 ± 0.0027	0.9927 ± 0.0024

**Table 7:** Ablation study on core components of RiMS-FiLM under fixed-budget risk screening (mean ± SD over five seeds).

Model	$p{AURC}_{0.30}$ ↓	Recall5	Recall10	Recall20	Recall30	Macro-F1 ↑	Micro-F1 ↑
w/o physical	0.1060 ± 0.0001	0.2375 ± 0.0000	0.4748 ± 0.0006	0.9420 ± 0.0026	0.9990 ± 0.0010	0.9859 ± 0.0014	0.9881 ± 0.0009
w/o prototype	0.1056 ± 0.0002	0.2375 ± 0.0000	0.4750 ± 0.0000	0.9473 ± 0.0021	0.9998 ± 0.0006	0.9908 ± 0.0030	0.9919 ± 0.0026
w/o conflict	0.1057 ± 0.0001	0.2375 ± 0.0000	0.4750 ± 0.0000	0.9463 ± 0.0027	0.9990 ± 0.0010	0.9910 ± 0.0027	0.9922 ± 0.0024
RiMS-FiLM	0.1056 ± 0.0001	0.2375 ± 0.0000	0.4750 ± 0.0000	0.9465 ± 0.0021	0.9995 ± 0.0007	0.9917 ± 0.0027	0.9927 ± 0.0024

## Data Availability

The maize subset of GrainSet used in this study is publicly available on Figshare (https://doi.org/10.6084/m9.figshare.22987562.v2). Project resources and baseline implementations are available at https://github.com/bimingwen/RiMS-FILM accessed on 1 October 2026.
